# Epidemiology of age-related macular degeneration (AMD): associations with cardiovascular disease phenotypes and lipid factors

**DOI:** 10.1186/s40662-016-0063-5

**Published:** 2016-12-22

**Authors:** Katie L. Pennington, Margaret M. DeAngelis

**Affiliations:** Department of Ophthalmology, John A. Moran Eye Center, University of Utah, Salt Lake City, UT USA

**Keywords:** Atherosclerosis, Hypertension, Retina, Statins, Stroke, Cholesterol, Obesity, BMI, Antioxidants, Genetic association

## Abstract

Age-related macular degeneration (AMD) is the leading cause of irreversible blindness in adults over 50 years old. Genetic, epidemiological, and molecular studies are beginning to unravel the intricate mechanisms underlying this complex disease, which implicate the lipid-cholesterol pathway in the pathophysiology of disease development and progression. Many of the genetic and environmental risk factors associated with AMD are also associated with other complex degenerative diseases of advanced age, including cardiovascular disease (CVD). In this review, we present epidemiological findings associating AMD with a variety of lipid pathway genes, cardiovascular phenotypes, and relevant environmental exposures. Despite a number of studies showing significant associations between AMD and these lipid/cardiovascular factors, results have been mixed and as such the relationships among these factors and AMD remain controversial. It is imperative that researchers not only tease out the various contributions of such factors to AMD development but also the connections between AMD and CVD to develop optimal precision medical care for aging adults.

## Background

Age-related macular degeneration (AMD) is a progressive blinding disease with no cure at present. In its most advanced stages, it deprives an individual of his or her ability to perform basic activities such as reading, recognizing faces, and driving. Approximately 11 million individuals are affected with AMD in the United States (U.S.) alone, with a global prevalence of 170 million. AMD is thereby the leading cause of visual disability in the industrialized world and the third leading cause globally [1–3]. Aging is the greatest risk factor; therefore, the prevalence of AMD in the U.S. is anticipated to increase to 22 million by the year 2050, while the global prevalence is expected to increase to 288 million by the year 2040 [1, 2]. In the U.S., the prevalence of AMD is similar to that of all invasive cancers combined and more than double the prevalence of Alzheimer’s Disease [1, 4]. This high prevalence leads to an annual $4.6 billion direct healthcare cost due to AMD in the U.S. [5]. As the aging population increases, this expenditure is likewise expected to increase proportionately.

AMD is a neurodegenerative disease that preferentially affects the macular (central) region of the retina, although the reason for this is not clearly understood. The disease is categorized into early, intermediate, or advanced stages based on the severity of symptoms, including the number and size of drusen accompanied by hyper- or hypopigmentary changes and the presence or absence of choroidal neovascularization. The yellowish lipid-rich, protein-containing drusen deposits accumulate between the retinal pigment epithelium (RPE) and Bruch’s membrane and are symptomatic of early disease. Drusen are considered the “hallmark” of AMD. The term “dry AMD” refers broadly to early or intermediate stages as well as a late stage referred to as geographic atrophy (GA). The advanced GA stage involves the loss of RPE and choroid in at least the macular region of the retina, which leads to a gradual loss of photoreceptors and central vision [6–8]. The term “wet AMD” refers to the advanced neovascular (or exudative) stage of the disease, which presents a more rapid loss of vision relative to GA. Neovascular AMD arises from the growth of abnormal blood vessels from the choroid into the normally avascular sub-RPE and sub-retinal regions [choroidal neovascularization (CNV)] [8, 9]. Although neovascular AMD represents a small proportion of total AMD cases, it accounts for the majority of blindness associated with AMD [10].

**Fig. 1:**
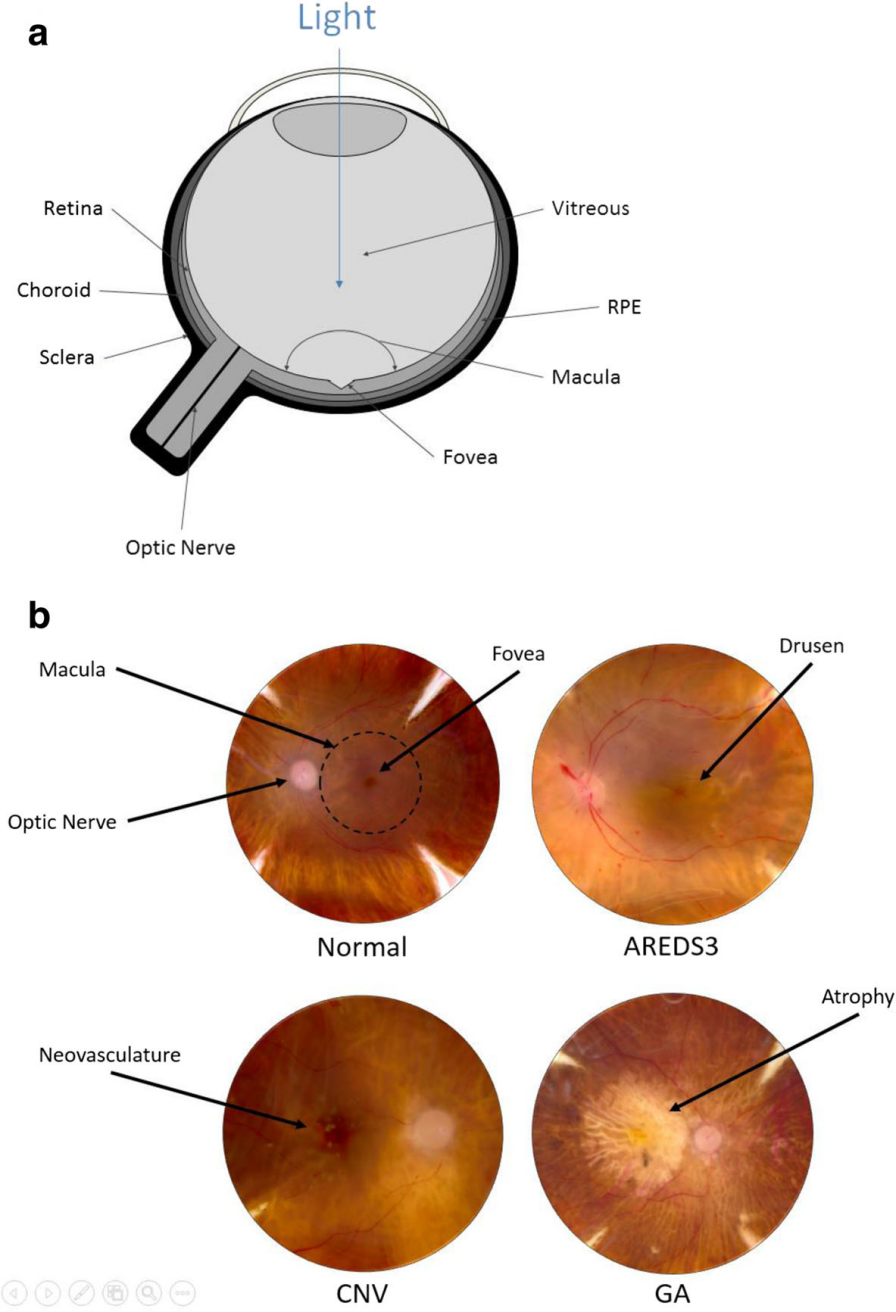
Progression of age-related macular degeneration. **a** Schematic drawing of an eye with relevant anatomic labels. **b** Fundus images of normal, intermediate (AREDS3), and advanced AMD (CNV and GA) eyes. Note the drusen deposits (AREDS3), atrophy (GA), and neovascularization (CNV). AMD, age-related macular degeneration; CNV, choroidal neovascularization; GA, geographic atrophy. Photos taken from DeAngelis lab patient cohorts. The study protocol was reviewed and approved by the Institutional Review Board at the University of Utah and conforms to the tenets of the Declaration of Helsinki

Precise diagnosis and staging requires an ophthalmic exam that includes fundus imaging of the retina for visualization of symptoms such as drusen deposits, pigmentary changes in the RPE, RPE and neural retinal degeneration and loss, and/or exudative changes in the retina (Fig. 1) [8, 11–13]. Further imaging with fluorescein angiography (which visualizes blood vessels) confirms the presence or absence of CNV [11]. Additional imaging techniques, such as optical coherence tomography (OCT), can also be implemented to confirm diagnosis. With these data, clinicians and researchers are able to categorize progression based on a standard grading scale, such as the Age-Related Eye Disease Study (AREDS) system, in which eyes are ranked on a scale of 1–4 [14, 15]. The AREDS grading system denotes non-AMD eyes as category 1 (AREDS1). Category 2 (AREDS2) eyes include early AMD cases in which symptoms were limited to small drusen <63 μm (also referred to as “hard” drusen), a single intermediate-sized druse 63–124 μm, and/or pigmentary changes. Category 3 (AREDS3) eyes include those with more extensive drusen such that they have at least one large druse >124 μm (“soft” druse), multiple intermediate drusen, and/or GA not involving the central macula. Category 4 (AREDS4) refers to eyes exhibiting GA involving the central macula and/or CNV. (See Khan et al. [16] for a detailed review of drusen and drusen-like deposits).

Most current therapies are directed toward the more advanced neovascular (“wet”) stage of AMD, target established abnormal blood vessel growth through antibody-based inhibition of vascular endothelial growth factor A (VEGFA), and demonstrate a range of efficacy. For a small subset of patients, these therapies result in stable to improved visual acuity without the need for ongoing treatment [17, 18]. However, the majority of patients require indefinite treatment or demonstrate progression of disease despite therapies [18]. With 1 in 10 people living in the U.S. aged 50+ years-old expected to be diagnosed with AMD by 2050 [19], affected individuals will continue to suffer and healthcare costs will rise exponentially unless the disease can be prevented, delayed, and/or treated effectively.

The development of new therapeutics, both for wet and dry forms of the disease, has received much attention, with the goal that they may ultimately improve outcomes and reduce the burden of treatment on affected individuals (see clinicaltrials.gov for a list of current clinical trials investigating a variety of potential therapeutics for both wet and dry AMD) [20, 21]. Therapeutics that can prevent the progression from early to intermediate and/or from intermediate to advanced stages of the disease are greatly needed to alleviate the profound detrimental impacts of vision loss; elucidation of the molecular mechanisms involved in the etiology and progression of AMD, taking into account evidence from gene expression, epigenetic, molecular, and biochemical studies to complement genetic epidemiological studies, will be necessary to accomplish this goal [22–25].

Understanding the relationships among diseases that may share overlapping pathophysiology and/or are co-morbid pathologies with AMD could help to uncover disease etiology in AMD. More importantly, because multiple co-morbidities are common among the aging population, understanding any commonalities in disease pathology among these various conditions will also improve the co-management of co-occurring conditions, such as AMD co-occurring with cardiovascular disease (CVD), allowing for synchronous preventative and/or therapeutic approaches.

AMD is a complex disease with many genetic and environmental factors, as well as interactions among these many factors, which influence susceptibility to risk [25–39]. Some of these epidemiological risk factors for AMD can be modified, and include body-mass index (BMI), smoking tobacco, diet, and blood lipid and cholesterol levels [35]. However, other factors cannot be modified at present, including genotype at a given risk locus, sex, ethnicity, and age.

## AMD pathogenesis

How these genetic and environmental factors influence the development and progression of disease remains largely unknown. One model for the development of advanced neovascular AMD suggests that the accumulation of drusen disrupts the connection between the RPE and the choroidal blood supply, thereby inducing hypoxia. The hypoxia in turn induces the expression of VEGFA and other pro-angiogenic factors to promote the formation of new vessels [40]. This model does not provide a complete picture, however, as extensive data support roles for local inflammation, complement activation, oxidative stress, and lipid homeostasis in the pathogenesis of AMD [24, 27, 41–45]. The accumulation of drusen deposits has been proposed to result from aberrant lipid influx into and efflux from the RPE [45, 46]. Likewise, drusen accumulation and composition appear to resemble atherosclerotic plaques [47]. Yet the exact molecular causes of AMD pathogenesis remain unclear. Although candidate gene, genome-wide association studies (GWAS), and epidemiological studies have implicated the lipid metabolism-cholesterol pathway in AMD pathophysiology, the role is unclear and at times inconsistent [24, 48–53].

## Drusen deposition and atherosclerotic plaque formation

Drusen are the hallmark lesions of AMD. The size and number of drusen deposits are generally indicative of disease severity and risk for progression to advanced disease [15, 54]. Studies investigating the composition of drusen have provided insight into the pathways involved in drusenogenesis and have illustrated commonalities with other degenerative processes such as atherosclerotic plaque formation (Table 1) [55]. Bruch’s membrane and the RPE function similarly to the blood brain barrier, with oxygen, lipids, and other nutrients passing between the choroidal blood supply and the retina via the RPE. As such, drusen lipid components appear to be sourced primarily from the RPE and photoreceptors, with the choroidal blood supply contributing a minor fraction, while the drusen proteins appear to come from both choroidal cells and serum [55]. This is in contrast to the fatty atherosclerotic plaque lesions in which the lipids, proteins, and lipoproteins are sourced systemically, that is, from the circulation [56]. Drusen and atherosclerotic plaques also have a number of components in common, which speaks to their common pathophysiologies, including complement components [i.e., vitronectin (VTN) and complement component 3 (C3)], amyloid (beta, P), apolipoproteins, esterified and unesterified cholesterol, matrix metalloproteinase (MMPs), and calcium [55, 56].

**Table 1:** Representative characteristics of drusen deposits and atherosclerotic plaques

Disease parameter	Drusen	Plaques	Reference/Reviewed in
Lipid deposits	X	X	Curcio et al. 2011 [203], Vaya 2013 [204]
Endothelial dysfunction	X	X	Machalinska et al. 2011 [205], Castellon & Bogdanova 2016 [206]
Complement activation	X	X	McHarg et al. 2015 [207], Vlaicu et al. 2016 [208]
Oxidative stress	X	X	FAnjul-Moles & Lopez-Riqelme 2016 [209], Vaya 2013 [204]
MMP involvement	X	X	Lambert et al. 2002 [210], Galis & Khatri 2002 [211]
Hyperhomocysteinaemia	X	X	Vine et al. 2005 [212]
Macrophage involvement	X	X	Grunin et al. 2014 [213], Chistiakov et al. 2015 [214]
Smooth muscle cell involvement		X	Chistiakov et al. 2015 [215]
Decreased blood flow	X	X	Berenberg et al. 2012 [216], Lowe 2003 [217]
VEGF upregulation/Progression with neovascularization	X	X	Bhutto & Lutty 2012 [218], Moreno et al. 2012 [219]
Rupture		X	Badimon & Vilahur 2014 [220]
Spontaneous regression	X		Toy et al. 2013 [221]
Lipid source	Local and circulating	Circulating	Booij et al. 2010 [56], Vaya 2013 [204]
Major deposit components			
Amyloid	X	X	Ohno-Matsui 2011 [222], Jans et al. 2006 [223]
Cholesterol/Cholesteryl esters	X	X	Pikuleva & Curcio 2014 [45], Vaya 2013 [204]
Lipoproteins	X	X	Curcio et al. 2011 [203], Linton et al. 2000 [224]

The “x” in columns 2 and 3 indicates that the parameter/component in the corresponding row is associated with/present in wither drusen (column 2) or atherosclerotic plaques (column 3)

Approximately 20 years ago, Dr. Friedman proposed the “hemodynamic model” of AMD after observing similarities between drusen and atherosclerotic plaques, which he updated over the following decade [57–60]. In this model, processes parallel to atherosclerotic plaque formation, leading from lipid deposition in the sclera and Bruch’s membrane to CNV were outlined. Friedman’s model hypothesized that the lipids deposited in the sclera increase scleral stiffness and choroidal vascular resistance, which decreases choroidal blood flow and increases choriocapillary pressure, which leads to CNV. Concurrently, lipid deposition in Bruch’s membrane leads to degeneration of elastin and collagen as well as basal deposits and drusen. The elastin and collagen degeneration leads to calcification, fracture, upregulation of VEGFA, and ultimately CNV. In this hypothesis, the lipid accumulation is the causative step leading to progression of disease. However, the drusen deposits themselves are proposed in this model to lead to RPE atrophy, but not to CNV, and would therefore not be causative of progression to neovascular disease.

Gelfand and Ambati recently published a “revised hemodynamic theory of age-related macular degeneration”, adding to this vascular model [37]. They propose that drusen selectively localize and accumulate as a result of local hemodynamic parameters within the choriocapillaris, which then determines the severity and progression of disease towards both GA and/or neovascular AMD. Although they incorporate the involvement of other processes, they posit that the hemodynamic factors are the initial and driving forces in AMD development and accumulation.

Additionally, a growing appreciation for the involvement of chronic inflammation, endothelial dysfunction, and oxidative stress in both AMD and atherosclerosis has shifted the emphasis of these diseases away from being considered simply lipid-deposition diseases [47]. Tan et al. argue in a recent review that immune system activation in AMD results from pathologic lipid accumulation [36]. However, Booij et al. contend that the accumulation of sub-retinal deposits is a normal process of aging, which only becomes pathogenic once the healthy involvement of the complement system becomes disrupted (likely through oxidative damage), uncontrolled, and thereby contributes to cellular damage and death [56]. Either way, the accumulation of drusen deposits precedes the further progression of pathological disease, although it has not been shown to be causative of progression [61–63]. Similarly, atherosclerotic plaques are thought to initiate from changes in the arterial endothelial cell lining, which when combined with dyslipidemia, hypertension, or pro-inflammatory agents, promotes the accumulation of low density lipoprotein- cholesterol (LDL-C) particles and further immune involvement leading eventually to smooth muscle migration, destabilization of the plaque, and ultimately thrombosis [64].

## Disease prevalence

The prevalence of AMD varies greatly by ethnicity with non-Hispanic White Europeans having the greatest disease burden. In this review, we refer to race and ethnicities as given by the authors of the original studies. We make no attempt to standardize the designations as each study has its own method for classifying participants.

A recent study by Wong et al. calculated pooled prevalence of ethnically diverse population-based studies of AMD (age range of 45–85 years) and confirmed that prevalence was greatest among those individuals of European descent at 12.3–30% with increasing age [2]. Disease burden, although slightly less, is still great amongst Hispanics (10.4%), Africans (7.5%) and Asians (7.4%) [2]. Still, others have estimated a lower disease burden within the U.S., with non-Hispanic White Europeans having the highest at almost 7.3% and African-Americans at 2.4% [65]. Regardless, it is clear that the prevalence of AMD varies by ethnicity and racial group, and therefore the role of genetic variants, environmental exposures, and their interplay in AMD susceptibility will likely vary by ethnicity as well.

## Genome-wide and candidate gene association studies of AMD

The lipid metabolism-cholesterol pathway has long been implicated in AMD, and emerged genetically in candidate gene and then GWAS studies [23, 50, 51, 66–68]. A recent exome chip study by Fritsche et al. identified 34 loci representing both common and rare variants near or around 860+ genes associated with AMD in individuals mostly of European Caucasian ancestry (Table 2) [24]. In addition to genes previously found at genome-wide significance, this study identified lipid pathway genes, including the *ATP-binding cassette transporter A-1* (*ABCA1*), *ABCA7*, *apolipoprotein C2* (*APOC2*), *APOC4*, and *phospholipid transfer protein* (*PLTP*); the complement pathway *VTN* gene; and the angiogenesis-associated *matrix metalloproteinase-9* (*MMP9*) gene. *MMP9* was shown specifically to be associated with the neovascular subtype and is the first gene to associate with a single AMD subtype. A number of other lipid associated pathway genes were previously shown by GWAS to associate with AMD, including genes encoding apolipoprotein E (APOE), cholesteryl ester transfer protein (CETP), and hepatic triglyceride lipase (LIPC) [68]. In fact, many of these genes were shown via molecular or candidate gene studies to be associated with AMD, including *ABCA1*, *ABCA7*, *APOE*, *CETP*, *LIPC*, and *MMP9* [48, 50, 51, 67, 69–77]. Several candidate gene studies have also implicated disease involvement for other lipid metabolism genes, such as *RAR related orphan receptor A* (*RORA)*, *roundabout guidance receptor 1(ROBO1)*, *lipoprotein lipase (LPL)*, *LDL receptor related protein 5 (LRP5)*, *LRP6*, *very low density lipoprotein receptor* (*VLDLR)*, *fatty acid desaturase 1–3* (*FADS1-3)*, and *adiponectin receptor 1* (*ADIPOR1)*, many of which have been implicated in populations of various ethnicities (including *RORA* and *ROBO1*) [49, 76–85]. (For further review, please see [45, 86]). The absence of genome-wide significant associations for these candidate genes may reflect the heterogeneous nature of AMD, the diversity of the populations, and the need for more rigorous and standardized phenotyping within the large, multi-center cohorts required for genome-wide analyses.

**Table 2:** Lipoprotein genes associated with AMD incidence and prevalence

Gene	Gene product	Chromosome	Function	Animal model	Association with AMD
ABCA1	ATP binding cassette subfamily A member 1	9q31.1	Cholesterol transport	KO [93, 225], conditional KO [94], transgenic [95]	Zareparsi et al. 2005 [69]
ABCA7	ATP binding cassette subfamily A member 7	19p13.3	Lipid homeostasis	KO [96]	Logue et al. 2014 [48]
APOE	Apolipoprotein E	19q13.2	Lipid catabolism	transgenic [97], KO [98]	Klaver et al. 1998 [67]
CETP	Cholesteryl ester transfer protein	16q21	Lipid metabolism	transgenic [99]	Chen et al. 2010 [50]
LIPC	Hepatic triglyceride lipase	15q21.3	Lipid metabolism	KO [100]	Chen et al. 2010 [50]
MMP9	Matrix metalloproteinase-9	20q13.12	ECM breakdown/angiogenesis	KO [101]	Hussain et al. 2011 [70]
PLTP	Phospholipid transfer protein	20q13.12	Lipid transfer to HDL	transgenic [102], KO [103]	Fritsche et al. 2015 [24]
VTN	Vitronectin	17q11	Cell adhesion/complement pathway	No	Fritsche et al. 2015 [24]

*ECM* = extracellular matrix; *HDL* = high-density lipoprotein; *KO* = knockout

One of the best characterized lipid-associated genes in AMD, *ABCA1* participates in cholesterol efflux out of tissues and into high-density lipoprotein (HDL) particles, catalyzing the rate limiting step of HDL particle formation and opposing atherosclerotic processes [87]. Impaired cholesterol efflux resulting from loss of *ABCA1* expression in older macrophages has recently been shown by Sene et al. to associate with the promotion of pathologic vascular proliferation in a mouse model of AMD [88]. Reduction of *ABCA1* expression was also observed in peripheral blood mononuclear cells in aging adult (67–87 years old) compared to young adult (25–34 years old) human donors [88]. DNA methylation at the *ABCA1* promoter has been shown to negatively correlate with HDL levels and incidence of coronary artery disease (CAD) in individuals with familial hypercholesterolemia, and *ABCA1* promoter methylation levels have been shown to increase with age [89, 90]. The accumulation of DNA methylation at the *ABCA1* promoter with age may account for the reduction of *ABCA1* expression observed by Sene et al. in older adults, which would potentially contribute to both increased risk of AMD and CAD in aging individuals. *ABCA1* agonists have been under study for their potential anti-atherogenic properties [91]. The work by Guay et al. and Sene et al. suggest that manipulating the *ABCA1* promoter methylation levels may be an alternative approach for increasing or restoring ABCA1 function and potentially reducing incidences of both CAD and AMD.

In addition to differences in disease prevalence among various ethnic and racial populations, recent work has also shown variability in the genetic contributions to disease risk among populations. The Population Architecture Using Genomics and Epidemiology (PAGE) study demonstrated that AMD risk appears to differ with respect to lipid metabolism and cholesterol-related genes in Mexican Americans, Asian Americans, African Americans, and non-Hispanic White Europeans when all types of AMD were examined [52]. In fact, in this study, none of the major risk variants for AMD e.g., *HtrA serine peptidase 1* (*HTRA1)/age-related maculopathy susceptibility 2 (ARMS2)* or *complement factor H* (*CFH*), were significant in the non-white European populations after correction for multiple testing, but this was likely due to the small sample size used in this study [52]. However, Cheng et al. found a novel variant in the *CETP* gene as well as novel AMD lipid/cholesterol genes associated with AMD risk in East Asians [92]. Moreover, the novel CETP risk variant was found to interact with high serum HDL levels in individuals of Japanese ancestry and Chinese from Singapore.

While GWAS and epidemiological studies have implicated the lipid metabolism-cholesterol pathway in AMD pathophysiology, the role is unclear and at times inconsistent [52, 53]. Given the variances within and among ethnic populations with respect to AMD genetics, prevalence, and pathology, further effort needs to be focused on working out the population-specific molecular mechanisms that may influence individual patient responses to therapeutic interventions and progression of the disease. To this end, transgenic and knockout animal models have been created to model some of these genes in vivo, including *ABCA1, ABCA7, APOE, CETP, LIPC, MMP9*, and *PLTP* (Table 2) [93–103].

## Epidemiology of cardiovascular health and AMD

Many recent studies have investigated the relationship between co-occurrences of AMD and cardiovascular-associated conditions, often with apparently contradictory findings (Table 3). An early paper by Kahn et al. in 1977 reported associations for AMD with systemic blood pressure and with left ventricular hypertrophy by combining data from participants in both the Framingham Heart Study collected from 1948–1964 and the Framingham Eye Study collected from 1973 to 1975 [104]. In the decades that followed, data for associations between various AMD phenotypes and a variety of cardiovascular-associated conditions and factors have amassed through an assortment of study designs. Age and tobacco smoking have been well-established as risk factors for both cardiovascular conditions and the development of AMD [105]. The following sections will highlight the studies presenting epidemiological associations among AMD phenotypes and other cardiovascular risk factors. Complicating factors in these studies include both the variety of cardiovascular-associated and AMD phenotypes used to represent risk or outcomes and the difficulty in achieving robust AMD phenotypes.

**Table 3:** Epidemiological studies investigating associations between AMD and cardiovascular/lipid conditions

Cardiovascular/Lipid Condition	Significant positive association found	Significant negative association found	No significant association found
CVD	Kahn et al. 1977 [104], Hyman et al. 1983 [117], Tan et al. 2007 [118], Fernandez et al. 2012 [124], Wu et al. 2014 [125]	Delcourt et al. 2001 [129], Nguyen-Khoa et al. 2008 [130]	Eye Disease Case–control Study Group 1992 [107], Klein et al. 1993 [108], Smith et al. 2001 [110], Klein et al. 2003 [109], Tomany et al. 2004 [111], Klein et al. 2007 [112], Fraser-Bell et al. 2008 [113], Golan et al. 2011 [116], Erke et al. 2014 [115]
Cardiovascular mortality	Clemons et al. 2004 [119], Tan et al. 2008 [137]		
MI	Duan et al. 2007 [121], Vassilev et al. 2015 [120]	Nguyen-Khoa et al. 2008 [130]	
CHD	Wong et al. 2007 [122], Sun et al. 2009 [123], Fernandez et al. 2012 [124], Wu et al. 2014 [125], Yang et al. 2014 [127]	Delcourt et al. 2001 [129]	
CAD	Thomas et al. 2015 [126], Wang et al. 2015 [128]		
Atherosclerosis	van Leeuwen et al. 2003 [132], Klein et al. 2007 [133], Taniguchi et al. 2015 [134]	Klein et al. 2003 [131]	
Stroke	Tan et al. 2007 [118], Fraser-Bell et al. 2008 [113], Tan et al. 2008 [137], Liao et al. 2008 [135], Hu et al. 2010 [136], Wieberdink et al. 2011 [138], Ikram et al. 2012 [139], Wu et al. 2014 [125]		Smith et al. 2001 [110], Delcourt et al. 2001 [129], Klein et al. 2003 [109], Tomany et al. 2004 [111], Klein et al. 2007 [112], Sun et al. 2009 [123], Erke et al. 2014 [115]
Hypertension	Kahn et al. 1977 [104], Sperduto & Hiller 1986 [144], Hyman et al. 2000 [145], Yang et al. 2014 [127], Cheung et al. 2014 [148], Thomas et al. 2015 [126]		Klein et al. 1993 [108], Smith et al. 2001 [110], Delcourt et al. 2001 [129], Klein et al. 2003 [131], Tomany et al. 2004 [111], Tan et al. 2007 [118], Fraser-Bell et al. 2008 [113], Yip et al. 2015 [147]
Diastolic blood pressure	Vidaurri et al. 1984 [143], Hyman et al. 2000 [145], Fraser-Bell et al. 2008 [113]		van Leeuwen et al. 2003 [132]
Systolic blood pressure	Klein et al. 2003 [109], van Leeuwen et al. 2003 [132], Erke et al. 2014 [115]		
Pulse pressure	Klein et al. 2003 [109], van Leeuwen et al. 2003 [132], Fraser-Bell et al. 2008 [113], Cougnard-Grégoire et al. 2013 [146]	Fraser-Bell et al. 2008 [113]	
Total serum cholesterol	Eye Disease Case–control Study Group 1992 [107], Tomany et al. 2004 [111], Ulaş et al. 2013 [149]	Klein et al. 1993 [108], Klein et al. 2003 [131], Tomany et al. 2004 [111], Cheung et al. 2014 [148]	Smith et al. 2001 [110], Delcourt et al. 2001 [129], Abalain et al. 2002 [151], Tan et al. 2007 [118], Munch et al. 2013 [153], Erke et al. 2014 [115], Mulero et al. 2014 [152], Klein et al. 2014 [53], Cheung et al. 2014 [148]
HDL-C	Klein et al. 1993 [108], Delcourt et al. 2001 [129], Klein et al. 2003 [109], Tomany et al. 2004 [111], Tan et al. 2007 [118], Cougnard-Grégoire et al. 2014 [150]		Smith et al. 2001 [110], Abalain et al. 2002 [151], Tan et al. 2007 [118], Erke et al. 2014 [115], Mulero et al. 2014 [152], Klein et al. 2014 [53], Cheung et al. 2014 [148]
LDL-C	Ulaş et al. 2013 [149]		Abalain et al. 2002 [151], Tan et al. 2007 [118], Erke et al. 2014 [115], Mulero et al. 2014 [152], Cheung et al. 2014 [148]
Cholesterol intake	Hyman et al. 2000 [145],		
HDL	Hyman et al. 2000 [145], Yip et al. 2015 [147]		Munch et al. 2013 [153]
LDL	Munch et al. 2013 [153]		
Total cholesterol/HDL-C ratio	Tan et al. 2007 [118]	Klein et al. 1993 [108]	Tan et al. 2007 [118], Klein et al. 2014 [53]
Hyperlipidemia	Vassilev et al. 2015 [120]		
Triglycerides	Munch et al. 2013 [153]		Delcourt et al. 2001 [129], Abalain et al. 2002 [151], Tan et al. 2007 [118], Munch et al. 2013 [153], Erke et al. 2014 [115], Mulero et al. 2014 [152], Yip et al. 2015 [147]
Phospholipids			Abalain et al. 2002 [151]
BMI	Klein et al. 2007 [112], Delcourt et al. 2001 [129], Ulaş et al. 2013 [149], Erke et al. 2014 [115]	Klein et al. 2003 [109], Klein et al. 2007 [112],	Smith et al. 2001 [110], Tomany et al. 2004 [111], DeAngelis et al. 2004 [154], Tan et al. 2007 [118], Munch et al. 2013 [153], Cheung et al. 2014 [148]
Physical exercise		Klein et al. 2003 [109], Munch et al. 2013 [153], Erke et al. 2014 [115]	
Waist circumference	Munch et al. 2013 [153]		Erke et al. 2014 [115]
Waist-to-hip ratio			Erke et al. 2014 [115]
Serum carotenoid levels		Eye Disease Case–control Study Group 1992 [107]	
Dietary lutein/zeazanthin		SanGiovanni 2007 [160], SanGiovanni et al. 2008 [161], Bonds et al. 2014 [163], Chew et al. 2014 [164], Wu et al. 2015 [162]	
Dietary DHA/EPA		SanGiovanni 2007 [160], SanGiovanni et al. 2008 [161], Wu et al. 2015 [162]	
Statin use	Klein et al. 2003 [173]	Hall et al. 2001 [165], McCarty et al. 2001 [166], Wilson et al. 2004 [167], McGwin et al. 2005 [168], Guymer at al. 2008 [175], Guymer et al. 2013 [176], Sasaki et al. 2013 [177], Ma et al. 2015 [174], Vavvas et al. 2016 [180]	Klein et al. 2007 [112], Cougnard-Grégoire et al. 2013 [146], McGwin et al. 2006 [170], Maguire et al. 2009 [171], Shalev et al. 2011 [114], Klein et al. 2014 [53], Al-Holou et al. 2015 [172]
Female Sex	Smith et al. 1997 [191], Rudnicka et al. 2012 [192]		Hirvelä et al. 1996 [189], AREDS Group 2000 [185], Buch et al. 2005 [184], Roh et al. 2008 [186], Laitinen et al. 2010 [183], Fong & Contreras 2010 [188], Chakravarthy et al. 2010 [190], You et al. 2012 [187]
Hormone therapy		Eye Disease Case–control Study Group 1992 [107], van Leeuwen et al. 2004 [194]	Smith et al. 2001 [110], Tomany et al. 2004 [111]
Age at menarche	Snow et al. 2002 [182]		
Age at menopause			Tomany et al. 2004 [111]
Time from menarche to menopause			Tomany et al. 2004 [111]

*CVD* = cardiovascular disease; *MI* = myocardial infarction, *CHD* = coronary heart disease; *CAD* = coronary artery disease; *HDL-C* = high-density lipoprotein cholesterol; *LDL-C* = low-density lipoprotein cholesterol; *HDL* = high-density lipoprotein; *LDL* = low-density lipoprotein; *BMI* = body-mass index; *DHA* = docosahexaenoic acid; *EPA* = eicosapentaenoic acid

### Cardiovascular disease

AMD phenotypes have been variably associated with a variety of CVD outcomes, including coronary heart disease (CHD)/CAD, myocardial infarction (MI), angina, or a pooled composite cardiovascular disease category [106]. Several studies have reported that they did not observe any association between AMD and cardiovascular disease, including reports from the Eye Disease Case–control Study Group [107], the Beaver Dam Eye Study (BDES, [108, 109]), and a pooled data set from the BDES, the Rotterdam Study, and the Blue Mountains Eye Study (BMES, [110]), each of which comprised a primarily white population. The 2004 evaluation of a pooled data set of the BDES, the BMES, and Rotterdam Study [111] focusing on incident GA, neovascular AMD, or any late AMD observed various significant associations within individual study groups for association with AMD, but observed no significant association for AMD with history of MI in the pooled dataset. A number of subsequent studies also reported no association between AMD and CVD, including the Women’s Health Initiative Sight Exam (WHISE) Ancillary Study [112], the Los Angeles Latino Eye Study (LALES, [113]), a report on a group of enrollees in a health maintenance organization in Israel [114], and the Tromsø Study [115].

However, several other studies have reported significant associations between AMD and CVD, representing associations between a variety of AMD subtypes/symptoms and cardiovascular outcomes for a variety of ethnic populations. Hyman et al. [116] and the BMES [117] reported significant associations between AMD and a history of CVD. The AREDS [118] and another BMES [119] found advanced AMD to associate with increased cardiovascular deaths, while the BMES (2008) also found early AMD to associate with increased CVD mortality.

Duan et al. [120] and Vassilev et al. [119] reported that AMD significantly associated with increased risk of myocardial infarction (MI). The Atherosclerosis Risk in Communities Study (ARCS, [121]) observed a significant association between late AMD and incident CHD in a population at high risk for CHD. However, in the Cardiovascular Health Study [122] comprised of a cohort of white Americans and African Americans, early AMD but not late AMD was associated with an increased risk of CHD. The baseline presence of AMD did not predict occurrence of CHD or CVD in the Multi-Ethnic Study of Atherosclerosis (MESA, [123]) full population, but late AMD did associate with both CVD and CHD in a subgroup comprised of older (65+) white participants. In a meta-analysis published in 2014 of eight prospective and five retrospective studies, Wu et al. [124] reported that patients with early AMD had an increased risk for CVD and for CHD. When they narrowed the analysis to include only the prospective studies, they also observed that subjects with late AMD had an increased risk for CVD. Thomas et al. [125] reported significant associations between AMD and CAD for individuals over 75 years old in a U.S. Veterans’ Affairs primarily male population.

Yang et al. [126] reported that rural Chinese subjects with CHD had increased risk of early AMD. Wang et al. [127] found that men with obstructive coronary stenosis are more likely to have early AMD and showed a correlation between the extent and severity of CAD and the prevalence of AMD.

In fact, other studies have reported inverse associations between AMD and cardiovascular outcomes, suggesting a protective effect, including data from the Pathologies Oculaires Liées à l’Age (POLA) Study [128] in which soft drusen were inversely associated with CHD, angioplasty, and any type of CVD. Also, Nguyen-Khao et al. [129] reported lower incidences of both MI and cerebrovascular accidents among patients with neovascular AMD compared to controls.

Continuation of the ongoing work on the processes underlying each of these conditions is critical to fully understanding the common biological factors predisposing individuals to each of these conditions and to allow for a more streamlined approach to managing diseases of older age.

### Atherosclerosis

Even with the apparent similarities between drusen and atherosclerotic plaques, associations for AMD with atherosclerosis are also inconsistent. Klein et al. observed no significant association between early AMD and common carotid artery plaques in a biracial U.S. population in the Cardiovascular Health Study reported in 2003 [130]. However, also in 2003, van Leeuwen et al. reported significant associations for AMD with having 4–6 plaques in the carotid artery and also with having a high composite score of atherosclerosis in the prospective Rotterdam Study [131]. Further, early AMD associated with echolucent carotid artery plaque in the full MESA study [132] cohort with other subclinical CVD factors having variable associations among the various ethnic subgroups. In addition, Taniguchi et al. [133] reported in 2015 that neovascular AMD associated with atherosclerosis.

The majority of these studies suggest a significant association between the presence of atherosclerotic plaques and the occurrence of AMD. This relationship is certainly complex and not likely to be a simple cause-and-effect scenario, but as researchers elucidate more of the molecular mechanisms contributing to each of these conditions, the precise details of their relationship will certainly emerge.

### Stroke

Many groups have reported data on the associations between stroke and AMD phenotypes, which have also presented conflicting data. Several studies have found no significant associations between AMD and stroke, including each of the following: the POLA Study [128]; pooled data from the BDES, the Rotterdam Study, and the BMES [110, 111]; the BDES [109] only; the WHISE Ancillary Study [112]; the Cardiovascular Health Study [122]; and the Tromsø Study [115].

However, several other papers have reported significant associations between AMD phenotypes and stroke. The LALES [113] reported that history of stroke or transient ischemic attack was associated with GA. Liao et al. [134] observed 2-year incident stroke cases in a cohort of 1.3 million Medicare enrollees with no major CVD at baseline, and observed significant associations between any AMD, neovascular AMD, and non-neovascular AMD with incident stroke, including both ischemic and hemorrhagic stroke. In a meta-analysis of eight prospective and five retrospective studies, Wu et al. [124] reported that subjects with late AMD had an increased risk for stroke. In 2012, Hu et al. [135] observed the 5 year incidence of stroke in a group of Taiwanese patients who received treatment for AMD, and observed an increased risk of stroke during the five-year follow-up period in patients with neovascular AMD compared to controls. Tan et al. [117] reported on the 10-year incidence of AMD associated with stroke from the BMES and observed significant associations for early but not late AMD, with a history of stroke. Later, Tan et al. [136] reported additional data from the BMES in which they found that among patients <75 years old at baseline, but not for patients 75+ years old at baseline, there was a tenfold increase in stroke mortality for patients with late AMD compared to controls without AMD. Wieberdink et al. [137] found that late AMD was significantly associated with an increased risk of any stroke and of intracerebral hemorrhage, but was not significantly associated with risk of cerebral infarction in the Rotterdam study. The ARCS [138] reported that subjects with either any or early AMD had increased risk of stroke, including both cerebral infarction and intracerebral hemorrhage.

As for the phenotypes discussed above, the specific stroke diagnostic phenotypes varied from study to study, which complicates the interpretation of the associations found between them and the various AMD phenotypes. However, the recurrence of such associations across studies suggests a relationship between the two conditions, thereby warranting closer inspection.

### Hypertension

Systemic hypertension has been shown to associate with decreased choroidal blood flow, which in turn is associated with the development of AMD, further suggesting that AMD development and/or progression have systemic contributions [139–141]. A number of studies have found significant associations between AMD phenotypes and blood pressure measures. As early as 1977, Kahn et al. reported associations between AMD and systemic blood pressure in data from the Framingham Eye Study [104]. Vidaurri et al. [142] reported an association between drusen and diastolic blood pressure in a Jewish population. In 1986, Sperduto & Hiller [143] reported a significant association between incidence of AMD and duration of hypertension, with longer duration of hypertension associated with higher risk of AMD. In 2000, Hyman et al. [144] reported significant associations for neovascular AMD with high diastolic blood pressure and hypertension in the AMD Risk Factors Study Group. The BDES [109] observed significant associations of higher systolic blood pressure with RPE depigmentation and neovascular AMD as well as higher pulse pressure with RPE depigmentation, retinal hyperpigmentation, neovascular AMD, and progression of AMD. Cougnard-Grégoire et al. [145] reported a significant association between elevated pulse pressure and risk of late AMD in the Antioxydants, Lipids Essentiels, Nutrition et maladies OculaiRes (ALIENOR) study in a French population. Yang et al. [126] reported that rural Chinese subjects with untreated hypertension had increased risk of early AMD. Thomas et al. [125] reported significant associations between AMD and hypertension in a U.S. Veterans’ Affairs primarily male population.

Other studies have not observed significant associations between AMD and blood pressure, including the BDES [108], the POLA study [128], a pooled BDES, BMES, and Rotterdam Study [110, 111], the Cardiovascular Health Study [130], the BMES alone [117], and the European Prospective Investigation Into Cancer (EPIC) Norfolk Eye Study [146].

A few papers reported inconsistent associations for different blood pressure measurements. The Rotterdam Study [131] observed significant associations for AMD with increased systolic blood pressure and increased pulse pressure, but no significant associations were found for diastolic blood pressure. In the LALES [113], no significant associations with AMD were observed for a history of hypertension, but significant associations were reported as follows: increased diastolic blood pressure associated with neovascular AMD; increased pulse pressure associated with RPE depigmentation and was protective for GA; and moderate pulse pressure was protective for neovascular AMD. Later, the Tromsø Study [115] showed significant associations for risk of late AMD with increased systolic blood pressure in women, but not in men. Cheung et al. [147] evaluated a multiethnic Asian population comprised of Chinese, Malay, and Indian subjects living in Singapore and reported a significant positive association for risk of early but not late AMD with hypertension.

As for the other cardiovascular-associated phenotypes, blood pressure measures are variably reported in these studies. Likewise, hypertension status can vary over an individual’s lifespan, thus confounding the relationship between measurements of blood pressure and AMD status.

### Lipid levels- triglycerides, cholesterol (HDL, LDL, total), phospholipids

The Eye Disease Case–control Study Group consortium [107] reported in 1992 that an increased risk of neovascular AMD was significantly associated with higher serum cholesterol levels. Similar to the other cardiovascular conditions, later studies showed mixed results regarding the association of serum cholesterol and triglyceride levels with AMD.

A large number of studies have shown significant associations between AMD and serum lipid levels. The AMD Risk Factors Study Group [144] observed a significant association for neovascular AMD with dietary intake of cholesterol and with high HDL levels. The biracial Cardiovascular Health Study [130] observed a significant association between early AMD and lower serum total cholesterol. The BDES (2013) reported a significant association between higher serum HDL- cholesterol (HDL-C) and pure geographic atrophy [109]. A pooled data set of the BDES, the BMES, and Rotterdam Study (Tomany et al. [111]) found significant lipid associations between total serum cholesterol levels positively associated with incident GA and inversely associated with incident neovascular AMD in the combined dataset. Ulaş et al. [148] reported that total cholesterol and LDL-C were significantly associated with neovascular AMD. The ALIENOR study [149] reported a significant association between elevated HDL-C levels and early or any AMD. Furthermore, Vassilev et al. [119] reported an increased risk of AMD for patients with hyperlipidemia.

Several other studies failed to show a significant association between AMD and serum lipid levels. Pooled data from the BDES, the BMES, and the Rotterdam Study [110] showed no significant associations with cholesterol or HDL-C. Abalain et al. [150] reported in 2002 on associations of AMD with serum lipids in a French cohort, and they observed no significant associations for AMD with cholesterol, triglycerides, phospholipids, HDL-C, or LDL-C. Data from the Tromsø Study [115] showed no significant association for late AMD with total cholesterol, LDL-C, HDL-C, or triglycerides. Mulero et al. [151] reported no significant association between neovascular AMD and total cholesterol, triglycerides, HDL-C, or LDL-C.

Yet, other studies reported variable associations among serum lipid levels and AMD. The BDES [108] found that in women, AMD related to low total serum cholesterol levels, but that in men, AMD was associated with both high HDL-C levels and a low total cholesterol/HDL-C ratio. The POLA Study [128] found that soft drusen were positively associated with HDL-C, but observed no significant association between AMD and total cholesterol or triglycerides. The BMES [117] observed significant associations for late AMD with HDL-C and the ratio of total cholesterol/HDL-C; but for early AMD, they observed no significant associations with HDL-C, LDL-C, triglycerides, total cholesterol, or the ratio of total cholesterol/HDL-C. Munch et al. [152] also observed an increased risk for moderate to large macular drusen in women with elevated levels of serum triglycerides, but no association was observed between moderate to large macular drusen and serum triglyceride levels in men. Triglyceride levels were also significantly associated in this study with the presence of 20+ small, hard macular drusen in both men and women with moderately elevated triglyceride levels, but not for subjects with the highest triglyceride levels. They further observed a significant increased risk for 20+ small, hard drusen for subjects in the second to lowest of five LDL levels compared to the lowest LDL level. They did not observe any associations between risk for 20+ small, hard drusen and HDL levels or total cholesterol. A meta-analysis of data from the BDES, BMES, and the Rotterdam Study (2014) did not observe significant associations between any of these measures and AMD outcomes in their combined analysis [53]. Cheung et al. [147] evaluated a multiethnic Asian population comprised of Chinese, Malay, and Indian subjects living in Singapore in which they observed a significant inverse association for risk of early AMD with total cholesterol. They did not observe any significant associations for late AMD with total cholesterol, LDL-C, or HDL-C. Yip et al. [146] observed a significant association between higher HDL levels and development of AMD in the EPIC Norfolk Eye Study, but found no association of AMD with serum triglyceride levels.

The underlying factors contributing to the observed associations or the lack of association between AMD phenotypes and lipid levels among various populations requires further investigation to understand their true relationships. Understanding the contributions of lipid factors to the development and progression of AMD will provide insight into the mechanisms of AMD pathology with the potential for presenting intervention options for the management of the disease.

### Obesity, BMI, & physical activity

Several studies have looked at the relationships among AMD-associated phenotypes and weight and physical activity. As for the data presented above for the other cardiovascular risk factors, reports on associations between AMD and weight/activity measures have also been mixed. The POLA Study [128], the WHISE Ancillary Study [112], and Ulaş et al. [148] reported significant associations for high BMI with increased risk for late AMD. The Cardiovascular Health Study [130] and the WHISE Ancillary Study [112] also found significant associations between lower BMI and incidence of GA. Further, the Cardiovascular Health Study [130] reported a significant protective association between physical exercise and the incidence of GA, neovascular AMD, and progression of AMD.

Alternatively, other studies were unable to identify significant associations for BMI with AMD. These include pooled data from the BDES, the Rotterdam Study, and the BMES [110, 111], BMES-only data [117], and a multiethnic Asian population comprised of Chinese, Malay, and Indian subjects living in Singapore [147], all of which reported no significant associations with BMI. DeAngelis et al. [153] reported data from extremely discordant sib pairs that were suggestive, but not significant, for association of BMI with neovascular AMD.

The results of other studies have presented mixed results, with differences arising between the sexes. The Inter99 Eye Study [152] reported a lower risk of moderate to large macular drusen associated with higher levels of physical activity for both men and women, but a differential association between waist circumference and risk for AMD that was differentiated by sex. In men, the likelihood of moderate to large macular drusen increased with increasing waist circumference, but for women, the likelihood of moderate to large macular drusen was higher for each the bottom and top quartiles of waist circumference. These authors did not observe any significant association between moderate to large macular drusen and BMI. Also, the Tromsø Study [115] found significant associations for risk of late AMD with high BMIs for women and a protective effect of exercise for women. However, these authors observed no significant associations for late AMD with waist circumference or waist-to-hip ratio in women and no significant associations were reported for any of the aforementioned factors for men.

Further studies are needed to work out the relationship of weight and activity factors with AMD, particularly given that weight and activity are both modifiable traits. This presents one difficulty in interpreting relationships between weight/activity measures and disease state in that these traits may vary substantially throughout an individual’s lifespan. However, if proven to be contributing elements to the development and/or progression of AMD, these factors would present interventional opportunities to help prevent or slow disease incidence or progression.

### Antioxidant and other supplement use

Oxidative stress has been associated with the development and progression of both AMD and CVD (reviewed in [154–157]). Antioxidants, including the long-chain omega-3 fatty acids, docosahexaenoic acid (DHA) and eicosapentaenoic acid (EPA), and the macular xanthophylls (MXs) lutein and zeaxanthin have been investigated for their potential roles in preventing the progression of AMD. Lutein and zeaxanthin are essential nutrients found in high quantities in the human retina [158]. In 1992, the Eye Disease Case–control Study Group consortium [107] reported a decreased risk for neovascular AMD significantly associated with higher levels of serum carotenoids (including lutein and zeaxanthin) in a U.S. population. This finding has been replicated by several studies, including the AREDS [159, 160] and the Nurses’ Health Study/ Heath Professionals Follow-up Study [161] which found inverse associations for dietary lutein/zeaxanthin and dietary DHA/EPA with AMD. The follow-up to the AREDS, AREDS2 [162], was a double-masked, randomized, controlled trial of nutritional supplementation looking at the effects on AMD progression, which found a significant effect of supplementation with lutein and zeaxanthin to reduce progression to neovascular AMD [162, 163]. However, the AREDS2 [162] also looked at the effects of lutein/zeaxanthin and DHA/EPA supplementation on CVD outcomes and reported no significant effect. The important role of MXs in this disease suggests a more complicated role for HDL in AMD than simply acting as a cholesterol carrier.

### Statin use

Hall et al. [164] initially reported a protective effect of statin (HMG-CoA reductase inhibitor) use on AMD in 2001, which was followed shortly by a report from McCarty et al. [165] similarly showing a protective effect of statin use. Likewise, Wilson et al. [166] showed in 2004 a significant retrospective association for statin use with decreased rates of CNV, and McGwin et al. [167] showed in 2005 a significant prospective association for cholesterol-lowering drug use and decreased risk of AMD. Guymer et al. [168] reviewed the various functions of statins in reducing atherosclerotic disease and proposed that such functions may also benefit AMD development, including the lipid-lowering, anti-inflammatory, and anti-angiogenic effects of statins. Specifically, they suggest that the statin-induced inhibition of high sensitivity C reactive protein and VEGFA expression may potentially contribute to reduced progression of AMD.

However, subsequent reports on associations for AMD and statin use have been predominantly negative for association. The Cardiovascular Health Study [169], the WHISE Ancillary Study [112], the Complications of Age-related Macular Degeneration Prevention Trial (CAPT) study [170], Shalev et al. [171], an ALIENOR study [145], and the AREDS2 [172] all observed no significant associations between AMD and statin use. A BDES report [173] indicated no significant association between statin use and AMD overall, but did report a significant association between those who started taking statins during the course of the BDES and the presence of large drusen or late AMD, although this association may have resulted from differences at baseline between those who initiated statins and those who did not. A meta-analysis of data from the BDES, the BMES, and the Rotterdam Study [53] observed significant associations between statin use with AMD outcomes within individual study groups; however, they did not observe significant associations between statin use and AMD outcomes in their meta-analysis. A meta-analysis [174] published in 2015 including 14 previously reported studies observed no significant association between statin use and any AMD. However, they did observe significant protective effects for statin use on both early AMD and neovascular AMD, but not for geographic atrophy.

The Age-Related Maculopathy Statin Study (ARMSS) reported in 2013 on a 3-year randomized, placebo-controlled double masked trial of simvastatin in an AMD population at high risk of progression to advanced disease [175–177]. They observed a reduction in the risk of progression of AMD with simvastatin treatment compared to placebo, as well as an increase in the retinal vascular caliber with treatment, both indicating a protective effect of statin use on AMD development. Patients with advanced AMD in one eye had no benefit of treatment on the risk of progression of the fellow eye to advanced disease. However, patients with bilateral intermediate AMD at baseline had a 77% reduction in risk of progression to advanced AMD compared to the placebo group. The authors also identified an interaction between response to simvastatin treatment and the Y402H risk allele of CFH such that patients homozygous for the C risk allele had a 12-fold reduction in AMD progression with simvastatin over patients with the heterozygous CT or homozygous TT alleles who also received simvastatin treatment. This study provides a strong justification for further investigation of statin use for the prevention of AMD progression, particularly in patients with intermediate bilateral AMD and/or those with the CHF Y402H CC risk genotype.

However, Gehlbach et al. [178] recently published a review of statin use for age-related macular degeneration in which they evaluated and compared the two random controlled trials of simvastatin for AMD to date (Guymer et al. [176] and Martini et al. [179]) and concluded that the current evidence is insufficient for the treatment of AMD with statins due to problems associated with each study. The Martini et al. trial was small and short (30 participants for three months), which was insufficient to properly evaluate either beneficial or adverse outcomes for this slowly progressing disease. Although the Guymer et al. study included 144 participants, only 70% of the participants completed the full 3-year follow-up.

Recently, Vavvas et al. [180] presented a case report in which a patient with dry AMD had regression of drusen following high-dose statin administration. This finding was followed up by a prospective, non-randomized interventional study in which 23 patients with early AMD and at high risk for disease progression were given high dose statin (80 mg atorvastatin) daily for up to 1.5 years. 10 of these patients experienced regression of drusen, and none of the patients progressed to advanced stage disease. This study included a narrowly defined risk group comprised entirely of Caucasian White U.S. and European patients who received a standard dose of a single statin. The authors propose that the heterogeneity within the study populations used to identify associations between statin use and AMD may account for the inconsistency of associations among prior studies. It would be interesting to see if a negative association for statin use with AMD progression or occurrence would be significant in existing data sets were they to be restricted to high risk individuals with early AMD who have taken high dose atorvastatin for 1 to 1.5 years. Further work with randomized, controlled interventional studies will be useful for working out the details and the extent to which this study can be extrapolated to patients that do not conform to the specific phenotypic and ethnic categories from which these study participants were drawn.

Certainly, more information is needed on the risks or preventive effects of statins, which are commonly being taken by aging patients. A better understanding of the relationship between these drugs and the development of AMD will improve the management of both cardiovascular disease and AMD. Further, any drugs that may prove to have a beneficial effect on AMD outcomes should be considered for their possible use as a therapeutic for the treatment of AMD.

### Sex

According to the National Eye Institute, 65% of prevalent AMD cases in 2010 were female patients [181]. One explanation for the higher prevalence of AMD among women is the longer life expectancy of women compared to men, which makes them more likely to acquire age-dependent diseases. This may not fully explain the sex differences observed for AMD, as some studies show differential risk associations for AMD among women compared to men. Further support for distinctions between the disease processes in men and women arise from the observations that the use of hormone therapies (HTs) have a protective effect against the development of AMD in women [182].

Epidemiological data has been mixed regarding the sex-associated risk of AMD. Many studies analyzing the sex-specific risk of AMD have not found significant associations between AMD and sex [183–190]. Other studies have shown differential disease risks for men and women [191, 192]. Munch et al. [152] reported in 2013 that they found a differential association between waist circumference and risk for AMD by sex. In 2014, Yang et al. [126] reported that, after adjusting for age and smoking status, men had a significantly increased risk of large drusen, but not early AMD and other specific lesions, suggesting a potential difference in disease pathology for men compared to women. Erke et al. reported on data from the Tromsø Study [115] in which they found significant associations for risk of late AMD with BMI, systolic blood pressure, and exercise only for women. A 2015 paper by Wang et al. [127] reported on a study group comprised of patients who presented to the hospital for assessment of suspected CAD, which was comprised of 76% men, in which they found risk for early AMD to be associated with cardiovascular risk factors differently for males than females.

Other studies have suggested a protective effect of estrogen on AMD development and/or progression. Snow et al. [182] observed an increased risk for AMD with older age at menarche [185]. Tomany et al. [111] evaluated a pooled data set of the BDES, the BMES, and Rotterdam Study and observed a significant association for older age at menopause with geographic atrophy in the BMES and a longer time between menarche and menopause associated with increased rates of GA in the Rotterdam Study. However, when the data from the three studies were pooled together, no significant associations between incident late AMD and age at menopause, time from menarche to menopause, or hysterectomy-induced menopause remained.

Current data also suggests a connection between AMD and the use of HT. Estrogen has antioxidant properties, which have been proposed to contribute to a protective role for AMD along with potential effects via the anti-inflammatory or other regulatory functions of estrogen [193]. Studies which identify a significant association between the use of HT and AMD suggest that these drugs may have a protective effect. In 1992, The Eye Disease Case–control Study Group consortium [107] reported a decreased risk for neovascular AMD significantly associated with the use of postmenopausal exogenous estrogens in a U.S. population. Later, both Smith et al. [110] in 2001 and Tomany et al. [111] in 2004 reported on pooled data from the BDES, BMES, and the Rotterdam Study where they observed no significant associations for incident late AMD with ever use of HT. However, van Leeuwen et al. [194] also analyzed a pooled dataset of information from the BDES, BMES, and the Rotterdam Study (2004) in which they observed a decreased risk of early AMD with use of HT in the pooled population. Note that this pooled analysis of the BDES, BMES, and Rotterdam Studies was performed contemporaneously with the pooled analysis by Tomany et al. [111]. However, the van Leeuwen analysis focused on incident early AMD in which participants with any AMD at baseline were excluded, whereas Tomany et al. evaluated incident GA, neovascular AMD, and combined late AMD regardless of the presence of AMD at baseline.

Cardiovascular disease is known to present differently in women compared to men, and the efficacy and side effects of treatments such as aspirin and statins may also vary by sex [195]. Estrogens may reduce the development of atherosclerotic plaques, but may also contribute to destabilizing existing plaques, thereby increasing cardiovascular events in the short term, but reducing these events in the long run [196–198]. Combining statin use with HT appears to prevent the estrogen-induced atherosclerotic plaque instability [199]. Combination statin/HT has also been observed to significantly decrease the risk of all-cause mortality (which was driven mainly by cardiovascular mortality) over statin-only treatment for primary prevention of cardiovascular events [200]. It will be interesting to determine whether a combined treatment plan including both a statin and HT would provide added benefit for the prevention of AMD progression.

The sex differences apparent for both AMD and CVD require that future studies are designed to maximize information for both sexes to fully understand both the commonalities and the differences underlying each of these diseases in both male and female patients. Along with considerations of genetic and environmental factors, sex needs to be considered in the design of personalized treatment regiments that optimize individual therapeutic responses. The recent NIH requirement to treat sex as a biological variable (see NIH notice NOT-OD-15-102 and related announcements) will improve reporting of data on sex-specific findings and further understanding of the sex differences inherent in this complex, heterogeneous disease. Even without individual studies being powered to detect sex differences, the availability of the data will allow for future meta-analysis to extract sex-specific findings.

## Conclusions

Given the complex and heterogeneous nature of both cardiovascular conditions and AMD, it would stand to reason that not all contributions to disease would be common to both conditions. For example, some genes associated with AMD [e.g., *complement factor I* (*CFI*), *TNF receptor superfamily member 10a* (*TNFRSF10A*), *beta 1,3-galactosyltransferase-like* (*B3GALTL*), and *solute carrier family 16 member 8* (*SLC16A8*)] have not to date been associated with CVD [24, 201]. However, given both the extent of epidemiological data, for both genetic and environmental factors, linking the diseases and the known molecular commonalities, there appears to be substantial overlap among the factors contributing to each condition. Understanding the relationship between AMD and CVD will certainly prove to be a significant advancement in our understanding of AMD, allowing the relatively young AMD field to benefit from prior and ongoing advances in the more mature cardiovascular field.

Clarifying the molecular, physiological, and pathological roles in AMD development and progression of each of the factors presented herein is necessary for a complete understanding of this blinding disease. Insights into the underlying molecular influences, including epigenetic influences (such as DNA methylation or histone modifications), on disease progression will also contribute to the development of improved therapeutic interventions. Not only do clinicians and scientists need to understand the various contributions of each factor, but understanding the interactions among risk/protective factors will also be essential to developing precision medicine in the clinical environment. A 2011 paper by Feehan et al. [202] divided neovascular AMD patients into four subtypes by both genetic and cardiovascular risk factors. In this analysis, the patients clustered based on blood pressure, hypercholesterolemia, BMI, and genotypes at the HTRA1 and CFH loci. Each cluster was defined by phenotype/genotype combinations, which illustrates the importance of looking across risk factors to understand each patient’s disease status and to design appropriate interventions. This study also underscores why many therapeutics fail during clinical trials, as they do not take both genotype *and* risk factor phenotypes into account simultaneously when examining disease outcome.

The epidemiological data presented herein must be validated clinically with randomized, double blind, prospective, interventional, controlled trials and biologically with molecular, cellular, and physiological studies in the laboratory to understand the complex pathophysiology and molecular mechanisms underlying the disease process. Such studies are critical for taking current understanding beyond observing disease associations to determining cause and effect relationships underlying disease, which will allow for targeted therapeutic intervention accordingly. Such interventions are crucial for improving the quality of life for millions of patients worldwide.

## Abbreviations

ABCA1: ATP-binding cassette transporter A-1
ABCA7: ATP-binding cassette transporter A-7
ADIPOR1: Adiponectin receptor 1
ALIENOR: Antioxydants, Lipids Essentiels, Nutrition et maladies OculaiRes
AMD: Age-related macular degeneration
APOC2: Apolipoprotein C2
APOC4: Apolipoprotein C4
APOE: Apolipoprotein E
ARCS: Atherosclerosis risk in communities study
AREDS: Age-related eye disease study
ARMS2: Age-related maculopathy susceptibility 2
ARMSS: Age-related maculopathy statin study
B3GALTL: Beta 1,3-galactosyltransferase-like
BDES: Beaver dam eye study
BMES: Blue mountain eye study
BMI: Body mass index
C3: Complement component 3
CAD: Coronary artery disease
CAPT: Complications of age-related macular degeneration prevention trial
CETP: Cholesteryl ester transfer protein
CFH: Complement factor H
CFI: Complement factor I
CHD: Coronary heart disease
CNV: Choroidal neovascularization
CVD: Cardiovascular disease
DHA: Docosahexaenoic acid
EPA: Eicosapentaenoic acid
EPIC: European prospective investigation into cancer
FADS1-3: Fatty acid desaturase 1–3
GA: Geographic atrophy
GWAS: Genome-wide association studies
HTRA1: Htra serine peptidase 1
LALES: Los Angeles latino eye study
LIPC: Hepatic triglyceride lipase
LPL: Lipoprotein lipase
LRP5: LDL receptor related protein 5
LRP6: LDL receptor related protein 6
MESA: Multi-ethnic study of atherosclerosis
MI: Myocardial infarction
MMP9: Matrix metalloproteinase-9
PAGE: Population architecture using genomics and epidemiology
POLA: Pathologies oculaires liées à l’age
ROBO1: Roundabout guidance receptor 1
RORA: RAR related orphan receptor A
RPE: Retinal pigment epithelium
SLC16A8: Solute carrier family 16 member 8
TNFRSF10A: TNF receptor superfamily member 10a
VEGFA: Vascular endothelial growth factor A
VLDLR: Very low density lipoprotein receptor
VTN: Vitronectin
WHISE: Women’s health initiative sight exam
